# Supplementary material for: The efficacy of resting-state fMRI denoising pipelines for motion correction and behavioural prediction
Source: Imaging Neurosci (Camb). 2025 Aug 7;3:IMAG.a.97. doi: 10.1162/IMAG.a.97 (PMC12336063; doi:10.1162/IMAG.a.97)
Supplement: Supplementary Material [file IMAG.a.97_supp.pdf]

The efficacy of resting-state fMRI denoising pipelines for motion correction and behavioural prediction.– Supplementary information

Kane Pavlovich, James Pang, Alexander Holmes, Toby Constable, Alex Fornito

Supplementary Methods

1. *Modularity Maximisation*

In the main text, we evaluated the performance of diffuse cluster estimation and regression (DiCER) using between 1 and 5 iterations. However, the same number of iterations is applied to all individuals, which may limit the performance of this algorithm. For instance, few iterations may be too lenient in individuals with high-noise data, whereas multiple iterations may be too aggressive in individuals with low-noise data (Aquino et al., 2020).

We developed a simply heuristic for tailoring the number of DiCER iterations to an individual dataset that is based on maximizing the modularity of the resulting functional coupling (FC) network. Modularity refers to the degree to which a network can be divided into cohesive communities, such that networks with high modularity have stronger FC within communities relative to a null model (Newman, 2006). In datasets with many wide-spread signal deflections (WSDs), FC networks are globally coherent and the network shows low modularity; similarly, if denoising is too aggressive, the network will show little structure, which also reduces network modularity. This implies that there may be an optimal number of DiCER iterations interposed between these two extremes that is associated with some maximal degree of modularity.

We used the Louvain algorithm (Blondel et al., 2008), as implemented in the Brain Connectivity Toolbox adapted for python v0.61 (Rubinov and Sporns, 2010) to find a partition of each person's FC network that seeks to maximize the Q-statistic, which quantifies the difference between intra-module FC and chance expectations,

$$Q = \frac{1}{2\mu}\sum_{i,j}\left(A_{ij} - \gamma V_{ij}\right)\delta\left(m_{i,}m_j\right), \tag{1}$$

where A_{ij} refers to the FC weight between nodes i and j, μ is the total weight of the network, $V_{ij} = \frac{k_i k_j}{2\mu}$ is the FC weight of the corresponding null model, with k_i and k_j corresponding to

the weighted degree of nodes i and j,. The Kronecker delta function $\delta(m_i, m_j) = 1$ if nodes i and j are in the same module and zero otherwise. The resolution parameter, γ, tunes the size of the resulting modules such that larger γ yields smaller modules.

The modularity maximization algorithm was run 100 times on individual FC matrices. The resulting 100 node assignments were then subjected to a consensus clustering (Lancichinetti and Fortunato, 2012) and the final Q value for a given FC matrix was computed from the consensus partition. This algorithm was executed across every iteration of DiCER, and the maximum Q value across iterations was chosen as the optimal number of DiCER iterations.

2. *Pipelines*

Supplementary Table 1. Different pre-processing pipelines employed in the our analysis.

Pipeline Number	*Pipeline steps*
1.	24P
2.	28P
3.	AROMA + 28P
4.	AROMA + 28P + Censoring
5.	FIX + 28P
6.	FIX + 28P + Censoring
7.	AROMA + 28P + GSR
8.	AROMA + 28P + GSR + Censoring
9.	FIX + 28P + GSR
10.	FIX + 28P + GSR + Censoring
11.	AROMA + 28P + DiCER
12.	AROMA + 28P + DiCER + Censoring
13.	FIX + 28P + DiCER
14.	FIX + 28P + DiCER + Censoring

3. *Behavioural Variables*

Supplementary Table 2. Behavioural variables used in the Kernel Ridge Regression (KRR) for the GSP dataset. For each variable, the corresponding field names in the GSP behavioural csv file are shown together with whether they belonged to the cognition or personality domain. The table has been adapted from Li et al. (2019).

Description	*Name in the GSP dataset*	*Cognitive*	*Personality*	*Others*
Flanker Accuracy	Flanker_S_CORRpc	✓		
Mental Rotation Accuracy	Average of MenRot_80_CORRpc, MenRot_120_CORRpc, and MentRot_160_CORRpc	✓		
Shipley Vocabulary	Shipley_Vocab_Raw	✓		
WAIS Matrix Reasoning	Matrix_WAIS	✓		
Speilberger Trait Anxiety	STAI_tAnxiety			✓
Speilberger State Anxiety	STAI_sAnxiety			✓
Total Mood Disturbance	POMS_TotMdDisturb			✓
Barratt Impulsivity	Barratt_tot			✓
Mind Wandering Frequency	MindWandering_Freq			✓
NEO Neuroticism	NEO_N		✓	
NEO Extraversion	NEO_E		✓	
NEO Openness	NEO_O		✓	
NEO Agreeableness	NEO_A		✓	
NEO Conscientiousness	NEO_C		✓	
Novelty Seeking	TCI_Novelty		✓	
Reward Dependence	TCI_RewardDependance			
Harm Avoidance	TCI_HarmAvoidance			✓
Risk Taking	DOSPERT_taking		✓	
Behavioural Activation Drive	BISBAS_BAS_Drive			✓
Behavioural Activation Fun	BISBAS_BAS_Fun			✓
Behavioural Activation Reward	BISBAS_BAS_Reward			✓
Behavioural Inhibition	BISBAS_BIS			✓

Supplementary Table 3. Behavioural variables used in the KRR for the HCP dataset. For each variable, the corresponding field names in the HCP behavioural csv file are shown together with

whether they belonged to the cognition or personality domain. The table has been adapted from Li et al. (2019).

Description	*Name in the HCP dataset*	*Cognitive*	*Personality*	*Others*
Visual Episodic Memory	PicSeq_Unadj_Score	✓		
Cognitive Flexibility (DCCS)	CardSort_Unadj	✓		
Inhibition (Flanker Task)	Flanker_Unadj	✓		
Fluid Intelligence (PMAT)	PMAT24_A_CR	✓		
Vocabulary (Pronunciatio n)	ReadEng_Unadj	✓		
Vocabulary (Picture Matching)	PicVocab_Unadj	✓		
Processing Speed	ProcSpeed_Unadj	✓		
Delay Discounting	DDisc_AUC_40K	✓		
Spatial Orientation	VSPLOT_TC	✓		
Sustained Attention – Sens.	SCPT_SEN	✓		
Sustained Attention – Spec.	SCPT_SPEC	✓		
Verbal Episodic Memory	IWRD_TOT	✓		
Working Memory (List Sorting)	ListSort_Unadj	✓		
Cognitive Status (MMSE)	MMSE	✓		
Sleep Quality (PSQI)	PSQI_Score			✓

Walking Endurance	Endurance_Unadj			✓
Walking Speed	GaitSpeed_Comp			✓
Manual Dexterity	Dexterity_Unadj			✓
Grip Strength	Strength_Unadj			✓
Odour Identification	Odor_Unadj			✓
Pain Interference Survey	PainInterf_Tscore			✓
Taste Intensity	Taste_Unadj			✓
Contrast Sensitivity	Mars_Final			✓
Emotional Face Matching	Emotion_Task_Face_Acc			✓
Arithmetic	Language_Task_Math_Avg_Difficulty_Level	✓		
Story Comprehension	Language_Task_Story_Avg_Difficulty_Level	✓		
Relational Processing	Relational_Task_Acc			✓
Social Cognition – Random	Social_Task_Perc_Random			✓
Social Cognition – Interaction	Social_Task_Perc_TOM			✓
Working Memory (N-back)	WM_Task_Acc	✓		
Agreeableness (NEO)	NEOFAC_A		✓	
Openness (NEO)	NEOFAC_O		✓	
Conscientiousness (NEO)	NEOFAC_C		✓	
Neuroticism (NEO)	NEOFAC_N		✓	
Extraversion (NEO)	NEOFAC_E		✓	

Emotion Recognition – Total	ER40_CR			✓
Emotion Recognition – Angry	ER40ANG			✓
Emotion Recognition – Fear	ER40FEAR			✓
Emotion Recognition – Happy	ER40HAP			✓
Emotion Recognition - Neutral	ER40NOE			✓
Emotion Recognition – Sad	ER40SAD			✓
Anger – Affect	AngAffect_Unadj		✓	
Anger – Hostility	AngHostil_Unadj		✓	
Anger – Aggression	AngAggr_Unadj		✓	
Fear – Affect	FearAffect_Unadj		✓	
Fear – Somatic Arousal	FearSomat_Unadj		✓	
Sadness	Sadness_Unadj		✓	
Life Satisfaction	LifeSatisf_Unadj			✓
Meaning & Purpose	MeanPurp_Unadj			✓
Positive Affect	PosAffect_Unadj		✓	
Friendship	Friendship_Unadj			✓
Loneliness	Loneliness_Unadj			✓
Perceived Hostility	PercHostil_Unadj			✓
Perceived Rejection	PercReject_Unadj			✓
Emotional Support	EmotSupp_Unadj			✓
Instrument Support	InstruSupp_Unadj			✓

Perceived Stress	PercStress_Unadj			✓
Self-Efficacy	SelfEff_Unadj			✓

Supplementary Figures

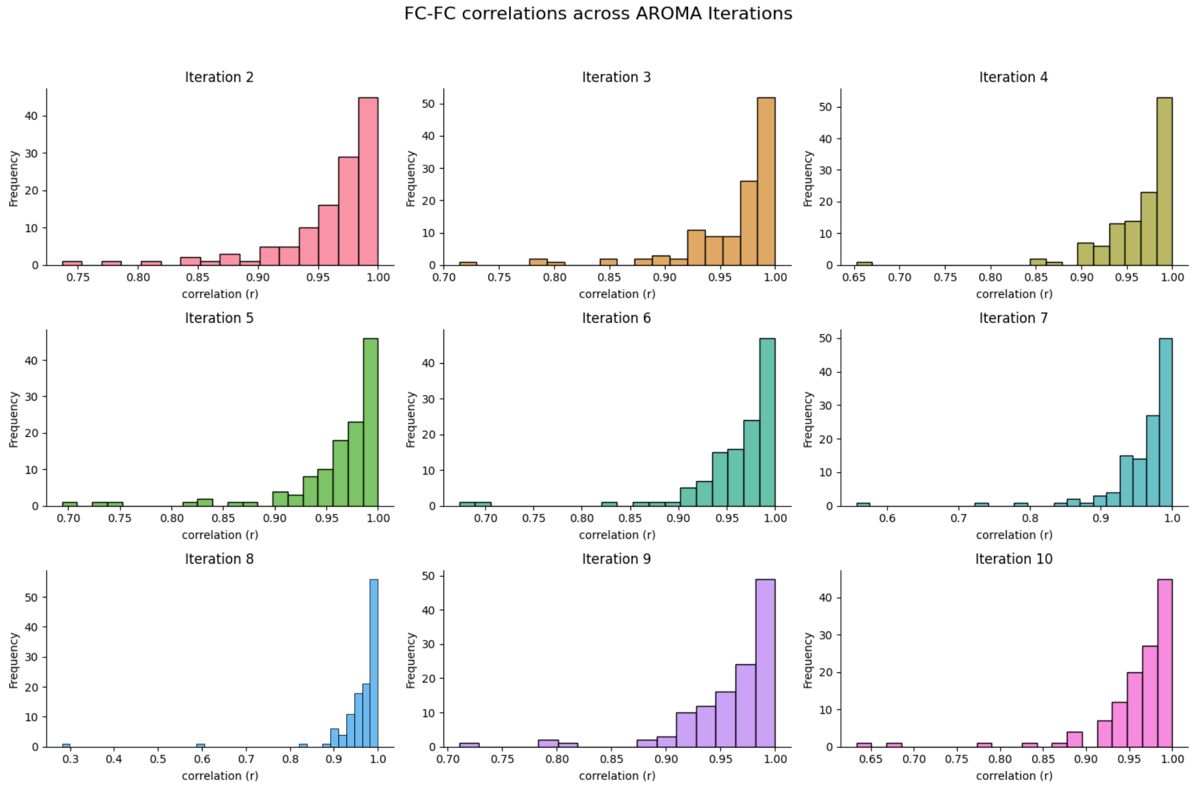

Supplementary Figure 1. Histograms illustrating the variability of ICA-AROMA across iterations in the CNP dataset. Each subject underwent a single ICA-AROMA iteration, where the upper triangle of their FC matrix was correlated against nine additional iterations using Pearson's R. The plot displays the nine FC-FC correlations for each subject, revealing the distribution of individual FC changes across different runs of ICA-AROMA.

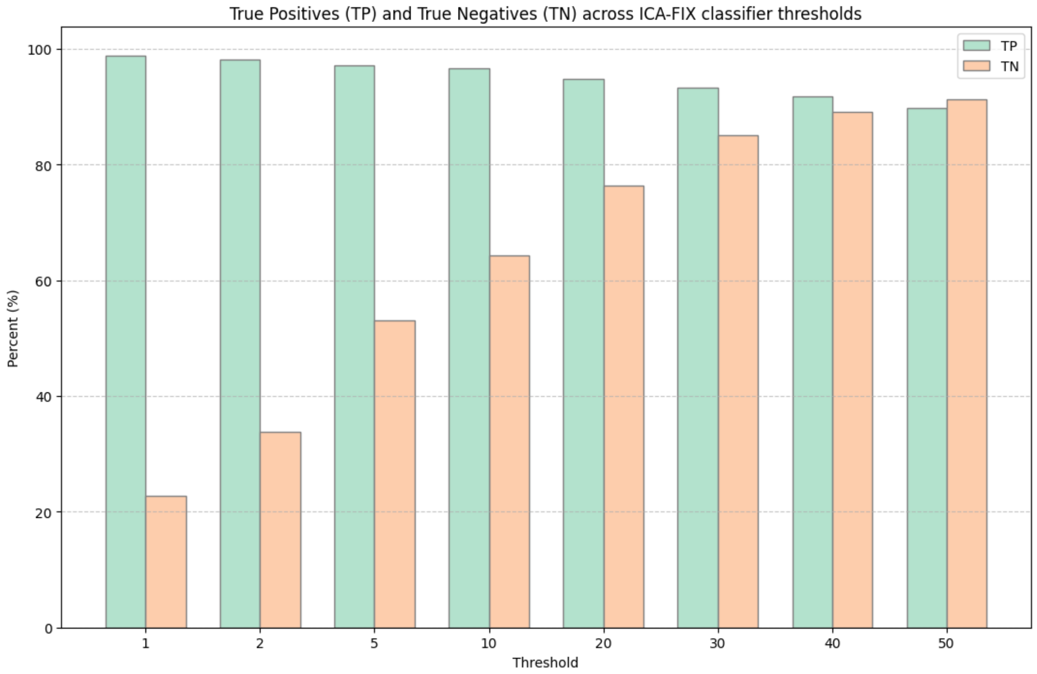

Supplementary Figure 2. Performance of the ICA-FIX classifier across various thresholds in the GSP dataset. Leave-one-out cross-validation was conducted on all training subjects, and the True Positive (TP) and True Negative (TN) rates were averaged across all participants for each classifier threshold. The final threshold for the test data was determined to be 40, as it optimized the rate of TN while simultaneously maintaining a high rate of TP.

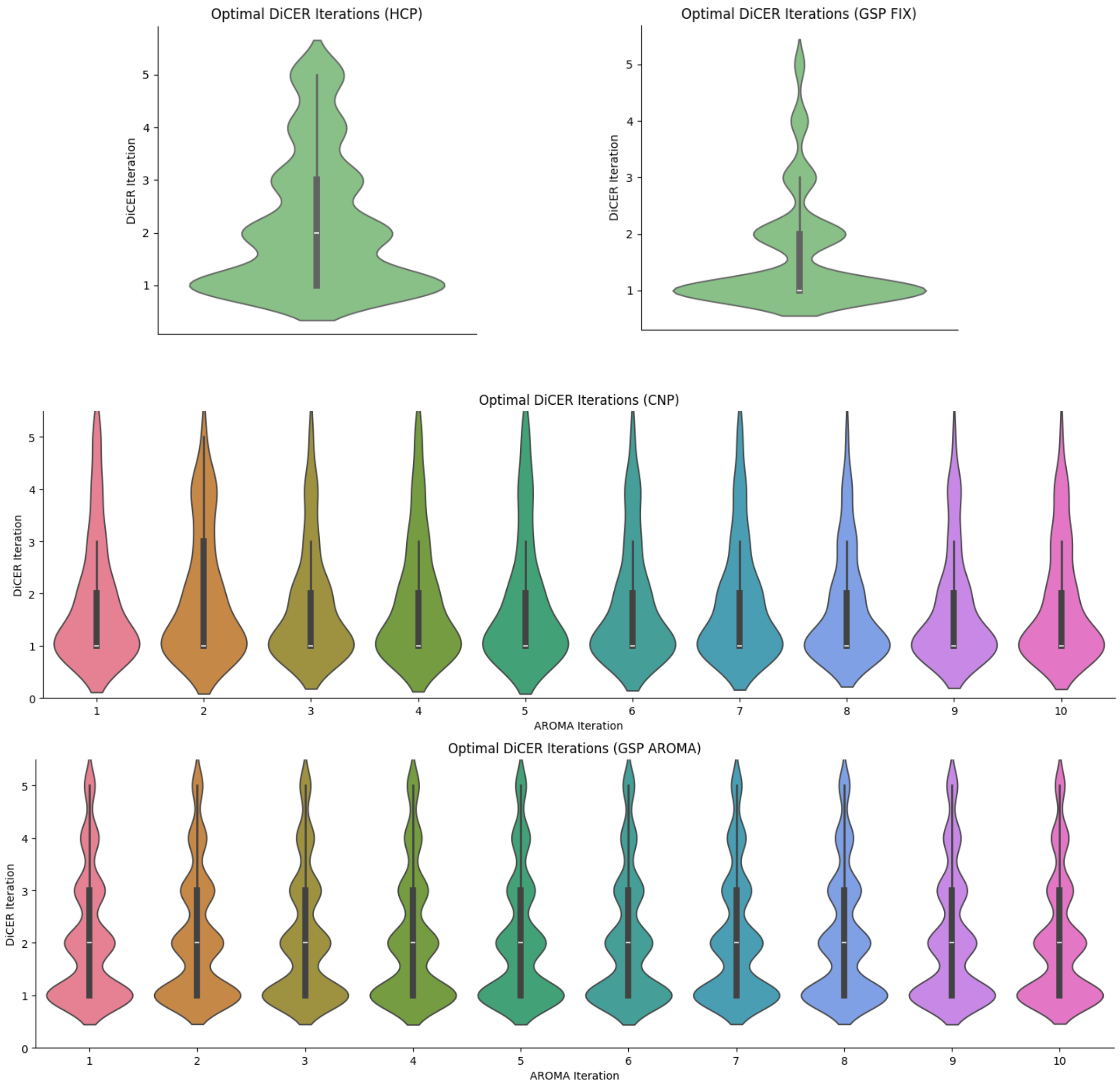

Supplementary Figure 3. Violin plots showing the number of iterations required by DiCER to achieve maximum modularity in each dataset. For pipelines utilizing ICA-AROMA, the maximum modularity was calculated separately for each run.

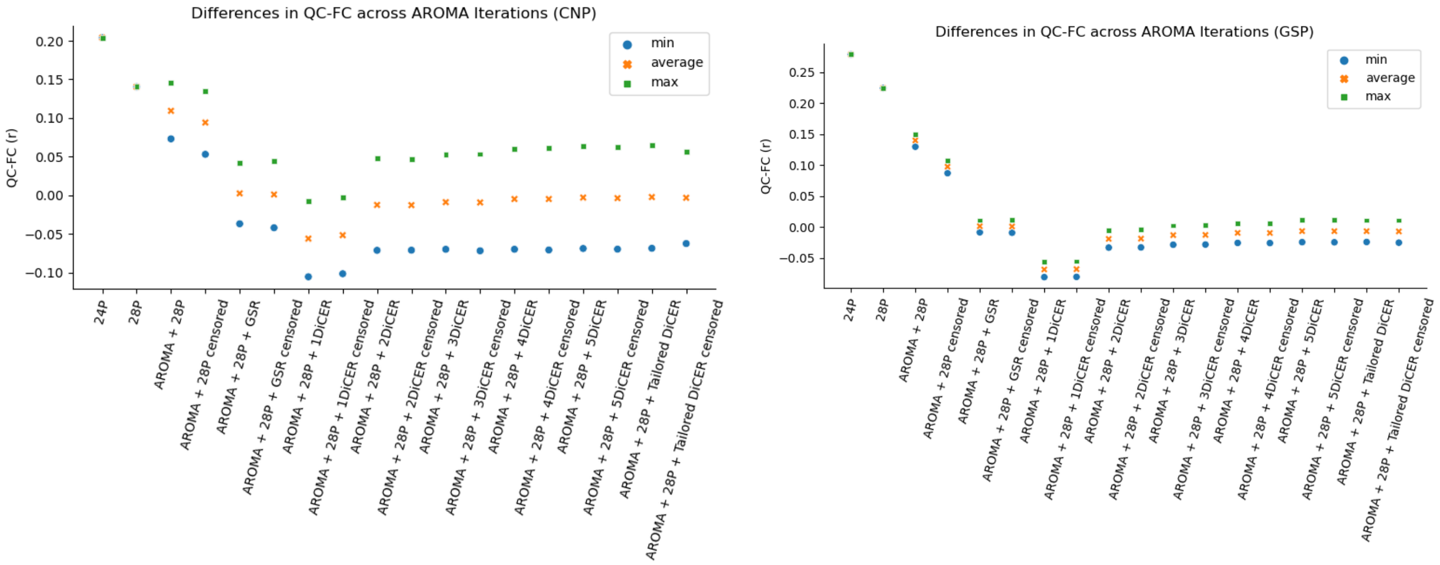

Supplementary Figure 4. Variability in QC-FC correlations across ICA-AROMA iterations. Scatter points depict the mean QC-FC value for each ICA-AROMA iteration, with distinctions for the lowest (blue dots), highest (green dots), and most average (orange stars) QC-FC results. The figure illustrates that differences observed across pipelines increase as more regressors are incorporated post ICA-AROMA.

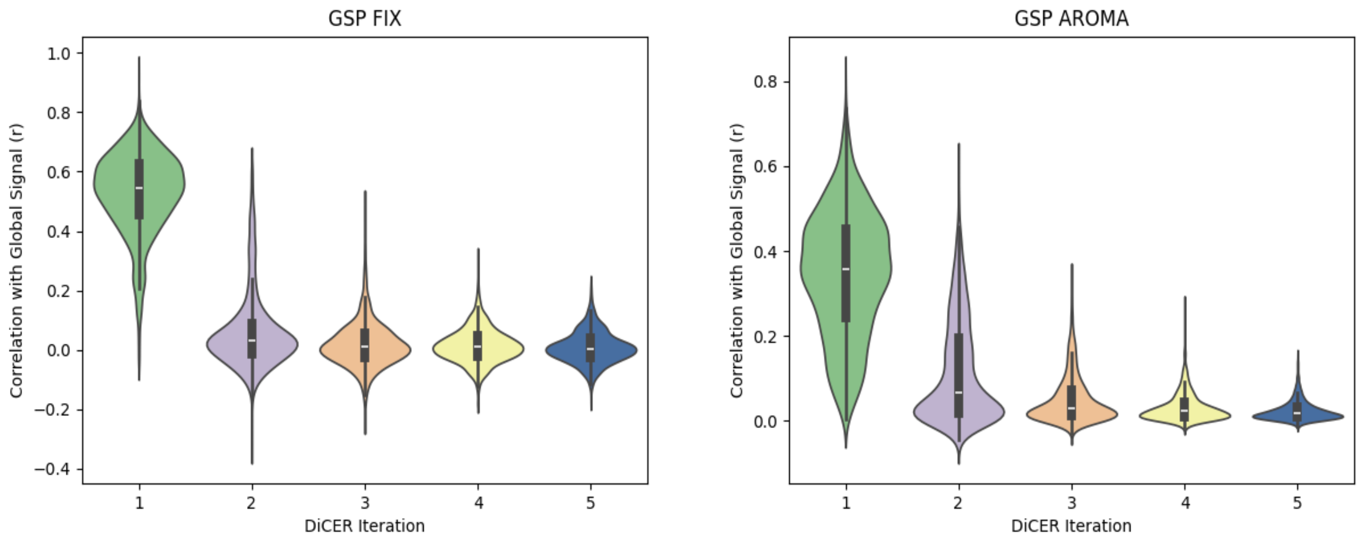

Supplementary Figure 5. Pearson's correlation of DiCER regressors with global signal in the GSP dataset. Violin plots depict the correlation between each subject's global signal and DiCER regressors. Shaded areas represent the kernel density estimate of each correlations distribution, while the boxes represent the median (white lines) and interquartile ranges. For pipelines incorporating ICA-AROMA, correlations were averaged across 5 iterations. The first DiCER regressor exhibits a moderate correlation with the global signal, the second displays a low correlation, while the subsequent regressors show no discernible correlation.

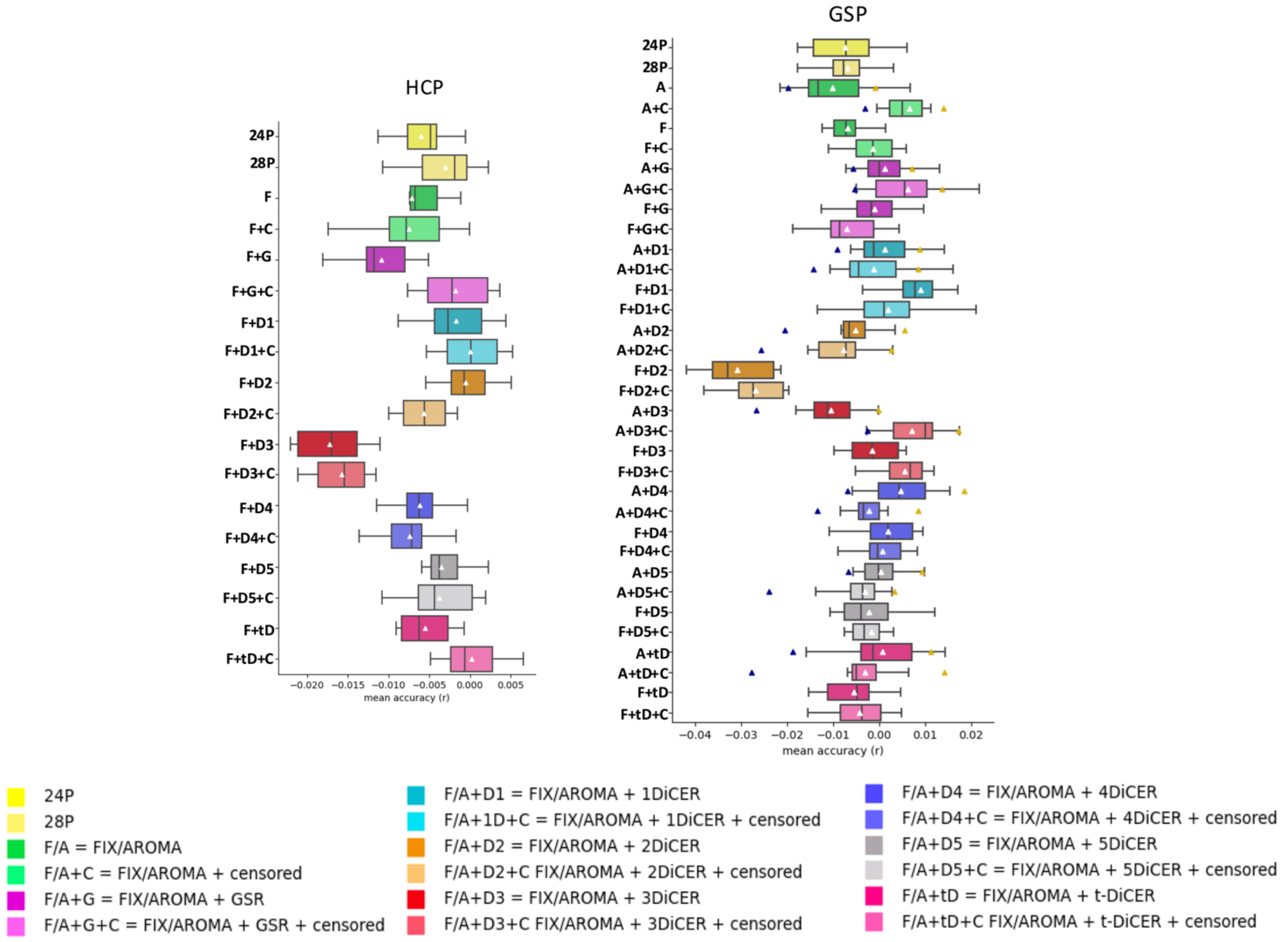

Supplementary Figure 6. Mean Kernel Ridge Regression (KRR) prediction accuracies averaged across all behavioural measures in the GSP and HCP datasets. Boxes depict the medians and interquartile ranges across 10 cross-validation splits and white triangles show the mean. For pipelines incorporating ICA-AROMA, the result shows an average across 10 different runs. Mean minimum performance across runs is represented by a blue triangle and mean maximum performance by a yellow triangle. Pipelines incorporating ICA-AROMA are labelled with two circles, while those using ICA-FIX are labelled with a square.

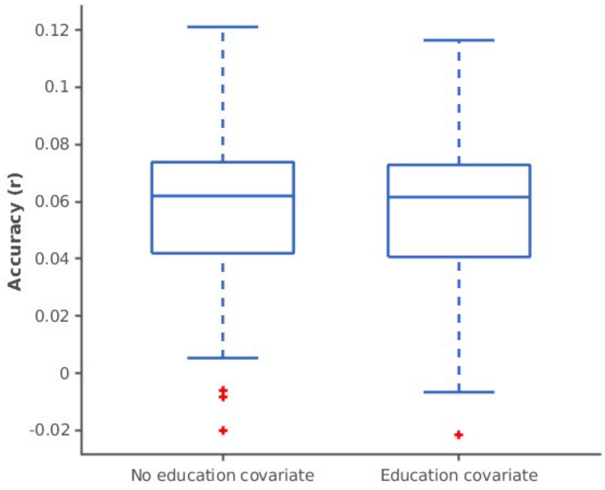

Supplementary Figure 7. Prediction accuracies averaged across 22 behaviours and 20 cross-validated folds for the best performing pipeline in the GSP dataset (ICA-FIX + 28P + 5-DiCER) with and without education as a covariate.

Supplementary Material References

Aquino, K. M., Fulcher, B. D., Parkes, L., Sabaroedin, K., & Fornito, A. (2020). Identifying and removing widespread signal deflections from fMRI data: Rethinking the global signal regression problem. NeuroImage, 212, 116614. https://doi.org/10.1016/j.neuroimage.2020.116614

Blondel, V. D., Guillaume, J.-L., Lambiotte, R., & Lefebvre, E. (2008). Fast unfolding of communities in large networks. *Journal of Statistical Mechanics: Theory and Experiment*, *2008*(10), P10008. https://doi.org/10.1088/1742-5468/2008/10/P10008

Lancichinetti, A., & Fortunato, S. (2012). Consensus clustering in complex networks. *Scientific Reports*, *2*(1), 336. https://doi.org/10.1038/srep00336

Newman, M. E. J. (2006). Modularity and community structure in networks. *Proceedings of the National Academy of Sciences*, *103*(23), 8577–8582. https://doi.org/10.1073/pnas.0601602103

Rubinov, M., & Sporns, O. (2010). Complex network measures of brain connectivity: Uses and interpretations. *NeuroImage*, *52*(3), 1059–1069. https://doi.org/10.1016/j.neuroimage.2009.10.003
